# Topical Melatonin Improves Gastric Microcirculatory Oxygenation During Hemorrhagic Shock in Dogs but Does Not Alter Barrier Integrity of Caco-2 Monolayers

**DOI:** 10.3389/fmed.2020.00510

**Published:** 2020-08-28

**Authors:** Richard Truse, Inga Nolten, Jan Schulz, Anna Herminghaus, Tobias Holtmanns, Lukas Gördes, Annika Raupach, Inge Bauer, Olaf Picker, Christian Vollmer

**Affiliations:** Department of Anesthesiology, Düsseldorf University Hospital, Düsseldorf, Germany

**Keywords:** gastric microcirculation, μHbO_2_, melatonin, hemorrhagic shock, Caco-2 monolayer, mucosal barrier integrity

## Abstract

Systemic administration of melatonin exerts tissue protective effects in the context of hemorrhagic shock. Intravenous application of melatonin prior to hemorrhage improves gastric microcirculatory perfusion and maintains intestinal barrier function in dogs. The aim of the present study was to analyze the effects of a topical mucosal melatonin application on gastric microcirculation during hemorrhagic shock *in vivo* and on mucosal barrier function *in vitro*. In a randomized cross-over study, six anesthetized female foxhounds received 3.3 mg melatonin or the vehicle as a bolus to the gastric and oral mucosa during physiological and hemorrhagic (−20% blood volume) conditions. Microcirculation was analyzed with reflectance spectrometry and laser doppler flowmetry. Systemic hemodynamic variables were measured with transpulmonary thermodilution. For analysis of intestinal mucosal barrier function *in vitro* Caco-2 monolayers were used. The transepithelial electrical resistance (TEER) and the passage of Lucifer Yellow (LY) from the apical to the basolateral compartment of Transwell chambers were measured. Potential barrier protective effects of melatonin against oxidative stress were investigated in the presence of the oxidant H_2_O_2_. During physiologic conditions topical application of melatonin had no effect on gastric and oral microcirculation *in vivo*. During hemorrhagic shock, gastric microcirculatory oxygenation (μHbO_2_) was decreased from 81 ± 8% to 50 ± 15%. Topical treatment with melatonin led to a significant increase in μHbO_2_ to 60 ± 13%. Topical melatonin treatment had no effect on gastric microcirculatory perfusion, oral microcirculation or systemic hemodynamics. Incubation of H_2_O_2_ stressed Caco-2 monolayers with melatonin did neither influence transepithelial electrical resistance nor LY translocation. Topical treatment of the gastric mucosa with melatonin attenuates the shock induced decrease in microcirculatory oxygenation. As no effects on local microcirculatory and systemic perfusion were recorded, the improved μHbO_2_ is most likely caused by a modulation of local oxygen consumption. *In vitro* melatonin treatment did not improve intestinal barrier integrity in the context of oxidative stress. These results extend the current knowledge on melatonin's protective effects during hemorrhage *in vivo*. Topical application of melatonin exerts differential effects on local microcirculation compared to systemic pretreatment and might be suitable as an adjunct for resuscitation of hemorrhagic shock.

## Introduction

Profound hemorrhage and associated hypovolemic shock are leading causes of potentially preventable deaths in the in-hospital and pre-hospital setting, resulting in annual deaths of more than five million people worldwide (1). During hemorrhagic shock, blood flow is redistributed early in favor of more vital organs (i.e., heart and brain) and splanchnic perfusion is usually impaired (2). Mesenteric hypoperfusion induces tissue hypoxia, ultimately causing organ failure. The local attenuation of microcirculatory oxygen supply reduces mucosal barrier function and has been shown to enable translocation of bacteria and endotoxins into portal venous blood and mesenteric lymph circulation, thereby mediating systemic inflammation (3). Maintaining adequate splanchnic perfusion, especially during circulatory shock is considered crucial for prevention and therapy of critical illness (4, 5). In this context melatonin is known to improve microcirculation and has been found in many tissues of the gastrointestinal tract. Melatonin is mainly released by the pineal gland at night, acting as the signal of darkness. However, exogenous administration of melatonin improves hepatic microcirculation and liver function and reduces stress-induced gastric lesions (6). In a previous investigation we were able to show that intravenous administration of melatonin prior to subsequent hemorrhage improves regional gastric microcirculatory perfusion during mild hemorrhagic shock in dogs and blunted the shock-induced damage to the intestinal barrier (7). However, whether melatonin exerts its protective impact via direct effects on the gastric mucosa, or via systemic effects remains unknown. Therefore, the objective of this study was to test whether the topical application of melatonin to the gastric mucosa may influence gastric microcirculation similar to systemic melatonin application in the context of hemorrhagic shock in dogs. As a pretreatment regime prior to a subsequent hemorrhagic shock in many cases does not reflect the clinical reality, in this setting the mucosal melatonin application was performed after onset of experimental hemorrhage. To further elucidate the protective properties of melatonin on gastrointestinal mucosal barrier function independent of microcirculatory changes, we analyzed its effects on the integrity of a Caco-2 cell monolayer during stress conditions.

## Materials and Methods

### Animals

The data were derived from repetitive experiments on six dogs (female foxhounds, 28–36 kg) treated in accordance with NIH guidelines for animal care and the ARRIVE guidelines. Experiments were performed with approval of the local animal care and use committee (North Rhine-Westphalia State Agency for Nature, Environment and Consumer Protection, Recklinghausen, Germany; reference number 84-02.04.2011.A288).

To exclude effects of the oestrus cycle, all dogs were castrated before inclusion into the study. Prior to the experiments, food was withheld overnight with water *ad libitum* to ensure complete gastric depletion and to avoid changes in perfusion and oxygenation due to digestive activity. Each dog underwent each experimental protocol in a randomized order and served as its own control (Supplementary Table 1). Experiments were performed at least 3 weeks apart to prevent carryover effects. The experiments were performed under general anesthesia (induction of anesthesia with 4 mg · kg^−1^ propofol, maintenance with sevoflurane, end-tidal concentration 3.0%, 1.5 MAC in dogs). The animals were mechanically ventilated after endotracheal intubation [F_i_O_2_ = 0.3, VT = 12.5 ml · kg^−1^, a normal tidal volume for dogs (8)] with the respiratory frequency adjusted to achieve normocapnia (end-expiratory carbon dioxide etCO_2_ = 35 mmHg). During baseline conditions, the dogs were placed on their right side and covered with warming blankets to maintain body temperature at 37.5°C. Throughout the experiments, no additional fluid replacement was administered to avoid volume effects that could influence tissue perfusion and oxygenation. However, after withdrawal of each blood sample for blood gas analysis and measurement of melatonin plasma concentration, normal saline was infused three times the sampling volume to maintain blood volume.

### Measurements

#### Systemic Hemodynamic and Oxygenation Variables

The aorta was catheterized via the left carotid artery for continuous measurement of mean arterial pressure (MAP, Gould-Statham pressure transducers P23ID, Elk Grove, IL) and intermittent arterial blood gas samples adjusted for temperature (Rapidlab 865, Siemens, Eschborn, Germany) from appropriate syringes (PICO50, Radiometer Medical, Brønshøj, Denmark). Cardiac output (CO) was determined via transpulmonary thermodilution (PiCCO 4.2 non US, PULSION Medical Systems, Munich, Germany) at the end of each intervention, at least every 30 min. Heart rate (HR) was continuously measured by electrocardiography (Powerlab, ADInstruments, Castle Hill, Australia). All hemodynamic and respiratory variables were recorded on a personal computer after analog to digital conversion (Powerlab, ADInstruments, Castle Hill, Australia) for later analysis.

#### Mucosal Oxygenation and Perfusion

Microvascular hemoglobin oxygenation (μHbO_2_) and perfusion (μflow) of the gastric and oral mucosa were continuously assessed by tissue reflectance spectrophotometry and laser Doppler flowmetry (O2C, LEA Medizintechnik, Gießen, Germany), as detailed previously (9). Briefly, white light (450–1,000 nm) and laser light (820 nm, 30 mW) is transmitted to the tissue of interest via a micro-lightguide and the reflected light is analyzed. The wavelength-dependent absorption and overall absorption of the applied white light can be used to calculate the percentage of oxygenated hemoglobin (μHbO_2_). Due to the Doppler effect, magnitude and frequency distribution of changes in wavelength are proportional to the number of blood cells multiplied by the measured mean velocity (μVel) of these cells. This product is proportional to flow and expressed in arbitrary perfusion units (aU). Hence, this method allows assessment and comparison of oxygenation and perfusion of the same region at the same time.

Since light is totally absorbed in vessels with a diameter >100 μm only microvascular oxygenation of nutritive vessels of the mucosa is measured. The biggest fraction of the blood volume is stored in venous vessels, therefore, mainly postcapillary oxygenation is measured which represents the critical partial pressure of oxygen (pO_2_) for ischemia (10).

One flexible light-guide probe is placed in the mouth facing the buccal side of the oral mucosa and a second probe is introduced into the stomach via an orogastric silicone tube and positioned facing the greater curvature. Both sites of measurement represent the microcirculation of different gastrointestinal mucosa regions (11). Online evaluation of the signal quality throughout the experiments allows verification of the correct position of the probe tip. The μHbO_2_ and μflow values reported are the means of the last 5 min (150 spectra, 2 s each) of the respective intervention under steady state conditions. The non-traumatic instrumentation and in particular non-traumatic access to the gastric mucosa allows the determination of mucosal microcirculation in the absence of surgical stress. This is particularly desirable with respect to the marked alterations that surgical stress exerts on splanchnic circulation (12). In this situation reflectance spectrophotometry reliably detects even clinically asymptomatic reductions in μHbO_2_ (13) and highly correlates with the morphologic severity and extent of gastric mucosal tissue injury (14).

#### Melatonin Plasma Levels

Plasma melatonin levels during baseline conditions were compared to plasma levels 30 min after topical melatonin administration. Plasma was collected in EDTA-Tubes (Vacutainer K2E EDTA 18.0 mg, Plymouth, UK) and stored at −80°C for later analysis. Plasma melatonin levels were assessed by means of enzyme-linked immunosorbent assay (ELISA) using commercially available kits (IBL International, Hamburg, Germany). Data were collected and analyzed according to the manufacturer's instructions and standards.

### Induction of Hemorrhagic Shock

Hemorrhagic shock was induced by removing 20% of the estimated total blood volume via a large bore intravenous catheter in a peripheral vein and the arterial catheter (i.e., 16 ml · kg^−1^ of whole blood over 5 min). According to Advanced Trauma Life Support this model represents a class II shock (15). This reversible and non-lethal shock model allows the investigation of either protective or harmful effects of various interventions, i.e., melatonin. Heparinized shed blood was stored and later retransfused using an infusion set with a 200 μm filter.

### Experimental Protocol

**Figure 1:**
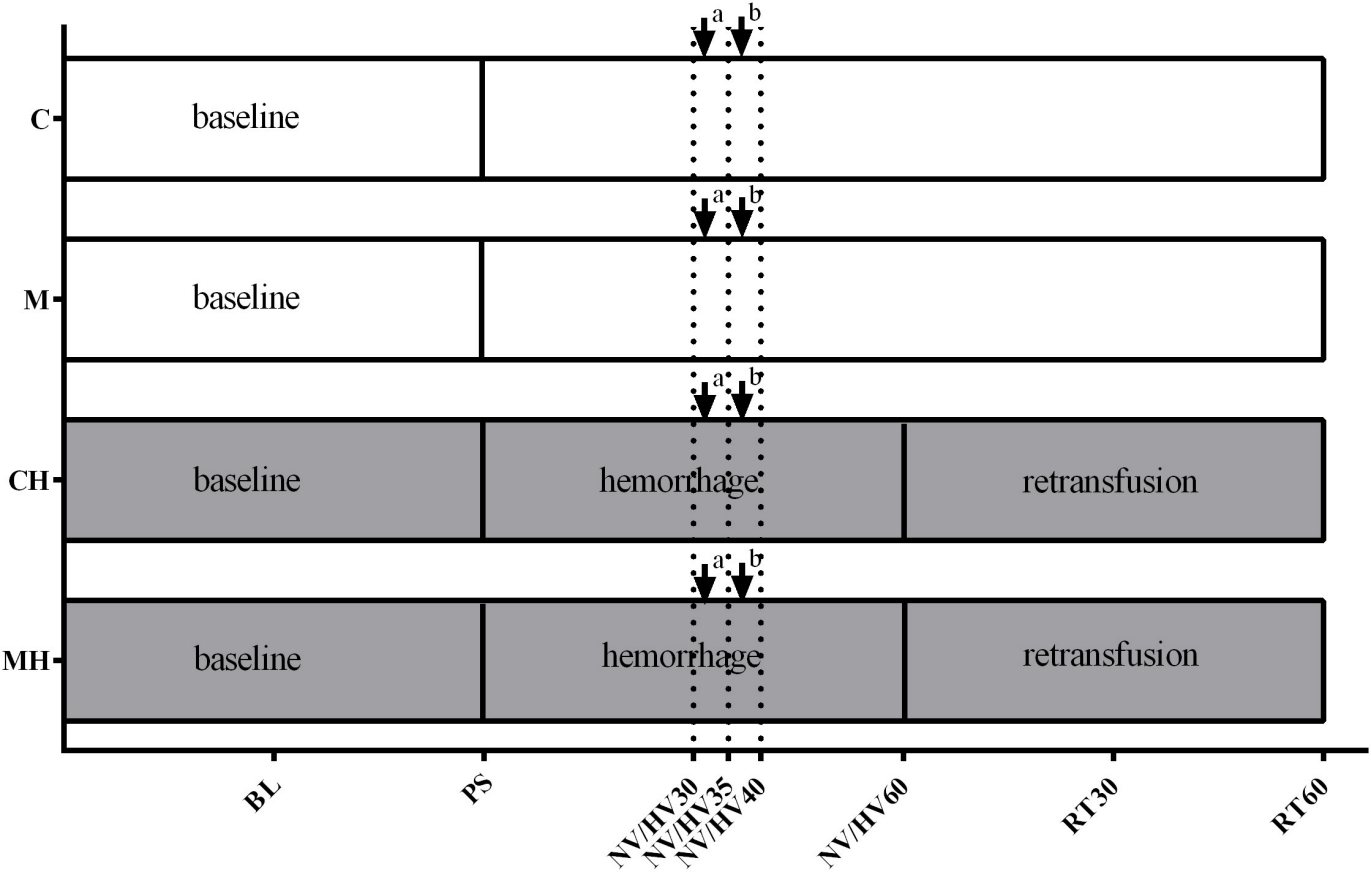
Experimental protocol. Experimental protocol: Application of the vehicle (C) or melatonin (M) during physiological conditions or 30 min after induction of hemorrhagic shock (CH, MH), ↓ bolus application to the oral and gastric mucosa, ↓^a^ melatonin 0.3 mg (M, MH)/NaCl 0.9% (C, CH); ↓^b^ melatonin 3 mg (M, MH)/ethanol 5% (C, CH). BL, baseline; PS, preshock; HV30/35/40/60 = 30/35/40/60 min after shock induction, NV30/35/40/60 = respective normvolemic control, RT30/60 = retransfusion.

Under steady state conditions baseline values were recorded before the animals were randomized to the respective protocol (Figure 1). Steady state conditions were defined as stability of hemodynamic variables (heart rate and mean arterial pressure) as well as ventilation parameters (endtidal CO_2_, endtidal sevoflurane concentration and inspiratory oxygen fraction). Content of the syringes (melatonin or vehicle) was blinded to the investigator during the experiments and data acquisition. Four different groups were analyzed:

#### Melatonin (Group M)

To study the effects of melatonin, in total 3.3 mg melatonin [Melatonin powder, Sigma-Aldrich, St. Louis, MO, USA; 0.3 mg melatonin diluted in NaCl 0.9% to a total volume of 3 ml, 5 min later followed by 3 mg melatonin diluted in 3 ml NaCl 0.9% containing 5% ethanol (Carl Roth, Karlsruhe, Germany)] was applied to the oral and gastric mucosa, and all variables were recorded for 2.5 h. Melatonin was applied next to the site of the microcirculation measurement. The gastric bolus reached the stomach via a gastric tube fixed to the O2C probe, whereas the bolus to the oral mucosa was applied around the measuring point via a perforated silicon tube. As melatonin is poorly soluble in water, we choose the solvent ethanol. Melatonin mediated effects on local microcirculation may be superseded by direct effects of ethanol. Therefore, melatonin was applied in two subsequent dosages with the lower concentration dissolved in NaCl 0.9% and the higher dissolved in ethanol. To assess early effects of melatonin on local microcirculation, microcirculatory oxygenation and perfusion were analyzed 5 min after each melatonin application.

#### Control Experiment (Group C)

As time control experiment, only the solvent (NaCl 0.9% followed by 5% ethanol solution, 3 ml) was applied topically to the oral and gastric mucosa, and all variables were recorded for 2.5 h.

#### Melatonin + Hemorrhagic Shock (Group MH)

To study the effect of melatonin on hemorrhage and retransfusion, after baseline measurements hemorrhagic shock was induced as described above and values were recorded for 30 min. Then melatonin was topically applied as described above and hemorrhagic shock was continued for further 30 min, followed by retransfusion of the shed blood with an additional observation period of 60 min.

#### Control Experiment, Hemorrhagic Shock (Group CH)

To study the effects of hemorrhage alone, only the solvent was applied during hemorrhage, followed by retransfusion as described above.

Every 30 min blood samples were obtained for blood gas analysis.

### Effects of Melatonin on Caco-2 Monolayer Integrity

Various confounders, like altered macro- and microcirculation, influence the intestinal barrier function *in vivo*. To separate potential direct effects of melatonin on barrier function from those mediated by improvement of microcirculation, we performed the analysis of the intestinal barrier function *in vitro*. We used Caco-2 cells grown on porous membranes as a model system of intact intestinal epithelium (16). These cells exert functional and anatomic similarities to absorptive intestinal enterocytes and hence this a well-established model to study intestinal permeability (17).

#### Reagents

Minimal essential medium eagle (MEME) was purchased from Sigma-Aldrich (Munich, Germany). Other cell culture reagents were obtained from Biochrom (Berlin, Germany) or life Technologies (Darmstadt, Germany).

#### Cell Culture

The human colon adenocarcinoma cell line Caco-2 (European Collection of Cell Cultures No. 86010202) was kindly provided by Dr. H. Steinbrenner (Institute for Biochemistry and Molecular Biology I, Heinrich-Heine-University Duesseldorf, Germany). Cells were cultured as described before (18) in MEME, containing 20% fetal calf serum, 1% non-essential amino acids, 1% L-alanyl-L glutamine-dipeptide (Glutamax) and 1% penicillin/streptomycin in a humidified atmosphere (5% CO_2_, 95% air, 37°C). The cells were grown under standard conditions until 60–70% confluency. Medium was changed three times a week.

#### Porous Membrane Supports

Porous membrane supports (Transwell-Clear, 12 mm diameter, 0.4 μm pore size, Corning Incorporated, NY, USA) were coated with 10 μg/cm^2^ collagen (Sigma-Aldrich, Munich, Germany). Then Caco-2 cells were harvested, seeded on the porous membrane inserts at a density of 2 × 10^5^ cells/cm^2^ and placed in a Transwell chamber. Cells were grown for 21–28 days to allow differentiation to build an intact, confluent layer, before experiments were performed.

#### Permeability Measurement

For permeability measurement of the Caco-2 monolayer the trans-epithelial electrical resistance (TEER) and the paracellular passage of Lucifer Yellow (LY, 444 Da, Thermo Fisher Scientific) from the apical to the basolateral compartment of the Transwell chamber were detected. All experiments were performed with *n* = 3 biological replicates, each assayed in duplicates.

Baseline TEER (Ωcm^2^) was measured using the EVOM2 Voltmeter (World Precision Instruments, Sarasota, FL, USA) with the Endohm2 chamber (World Precision Instruments, Sarasota, FL, USA) according to manufacturer's instructions. The unit “area resistance” is obtained by multiplying the meter readings by the effective surface area of the filter membrane. The dimension is Ωcm^2^. Values higher than 500 Ωcm^2^ were considered as sufficient confluence (19). After detection of baseline TEER, the medium was removed, and the inserts were incubated with 500 μl of the test component in the apical compartment and 1,000 μl of medium in the basal compartment for the determined period of time. Thereafter TEER (Ωcm^2^) measurement was repeated. Then medium was replaced with freshly prepared Hank‘s balanced salt solution adjusted to pH 7.4 for 15 min in non-CO_2_ atmospheric conditions. LY (200 μg/ml) was administered to the apical compartment. After 2 h LY concentration was measured in both compartments using a fluorescence spectrophotometer (excitation wavelength: 485 nm, emission wavelength: 535 nm). LY translocation was determined by calculating the ratio of basolateral to apical LY concentration as marker of barrier function.

#### Effect of Melatonin on Barrier Function

To test the effect of melatonin on TEER and LY translocation, damage was induced by the oxidant H_2_O_2_ [10 and 30 mM, incubation for 4 h, based on pilot experiments (Supplementary Figure 1)]. In a first set of experiments melatonin (0.1 mg/ml) was administered into the apical and basolateral compartment of the Transwell chamber diluted in the Caco-2 cell culture medium (see above) simultaneously with the administration of H_2_O_2_ and incubated for 4 h. In another set of experiments melatonin was added to the chamber 30 min prior to H_2_O_2_ followed by incubation for further 4 h.

### Statistical Analysis

Primary endpoints were defined as gastric/oral microcirculation *in vivo* and changes in LY translocation and TEER *in vitro*. Secondary endpoints were defined as systemic hemodynamic and oxygenation variables and plasma melatonin levels. All data are presented as mean ± standard deviation (mean ± SD). Statistical differences were analyzed with GraphPad Prism version 6.03 for Windows, GraphPad Software, La Jolla California USA. In the *in vivo* experiments a repeated measures two-way ANOVA followed by Dunnett's multiple comparisons test (*in vivo* experiments, vs. HV30) or Sidak *post hoc* test (*in vivo* experiments, vs. respective control group) were used. An *a priori* power analysis (G^*^Power Version 3.1.9.2) revealed a power of 0.85 for detection of differences between the different groups with *n* = 6 in four groups, repeated measurements, α < 0.05 and η^2^ of 0.5 (calculated from previous experiments). In the *in vitro* experiments the Kruskal-Wallis test followed by Dunn's multiple comparison test (non-parametric data) was performed. *p* < 0.05 was considered significant.

## Results

### Effects of Melatonin Under Physiological Conditions (Groups C and M)

Under physiological conditions, melatonin had no effect on gastric and oral μHbO_2_ and μflow (Table 1). During baseline conditions, gastric μflow was lower in group M compared to the control group. This effect was no longer evident in the further course of the experiment. Systemic hemodynamic variables remained stable throughout the experiment without differences to the control group. Microcirculatory and systemic variables are presented in Tables 2, 3, respectively.

**Table 1:** Microcirculatory variables.

**Parameter**	**Group**	**BL**				**PS**				**NV/HV30**				**NV/HV35**				**NV/HV40**				**NV/HV60**				**RT30**				**RT60**			
gastric μHbO_2_ [%]	C	77	±	3		80	±	2		79	±	7		79	±	6		75	±	3		77	±	4		74	±	6		74	±	7	
	CH	79	±	8	*	81	±	8	*	51	±	14		56	±	14		58	±	13		56	±	12		78	±	14	*	79	±	9	*
	M	77	±	6		77	±	7		76	±	7		77	±	6		76	±	8		76	±	7		75	±	8		76	±	8	
	MH	81	±	8	*	84	±	6	*	50	±	15		60	±	13	*	60	±	16	*	59	±	13	*	81	±	6	*	83	±	6	*
gastric μflow [aU]	C	120	±	39		117	±	26		122	±	14		117	±	14		112	±	11		100	±	37		102	±	28		104		33	
	CH	98	±	21	*	93	±	15		69	±	7		76	±	11		72	±	9		77	±	9		105	±	11	*	102		37	*
	M	86	±	42	**#**	112	±	30		106	±	41		119	±	31		118	±	35		108	±	44		115	±	40		111		37	
	MH	118	±	33	*	130	±	28	#*	83	±	36		91	±	31		91	±	40		96	±	35		118	±	28	*	123		33	*
gastric μvel [aU]	C	17	±	2		16	±	2		16	±	1		16	±	1		15	±	0		16	±	2		16	±	2		16		2	
	CH	16	±	2		15	±	2		14	±	2		14	±	1		14	±	1		14	±	1		16	±	1		16		4	*
	M	15	±	4		17	±	3		16	±	3		17	±	2		17	±	2		16	±	3		17	±	3		17		3	
	MH	18	±	4	#*	19	±	4	#*	15	±	3		15	±	2		15	±	3		16	±	3		16	±	1		18		4	#*
oral μHbO_2_ [%]	C	83	±	6		81	±	6		81	±	5		83	±	4		83	±	4		84	±	4		85	±	5		85	±	4	
	CH	85	±	5	*	83	±	2	*	48	±	6		54	±	5	*	57	±	5	*	57	±	7	*	90	±	6	*	95	±	2	*
	M	83	±	7		81	±	6		81	±	5		83	±	4		84	±	4		84	±	4		84	±	5		85	±	5	
	MH	84	±	6	*	82	±	4	*	47	±	6		51	±	7		54	±	10	*	57	±	7	*	90	±	6	*	92	±	6	*
oral μflow [aU]	C	169	±	49		158	±	53		157	±	55		161	±	57		164	±	48		173	±	60		175	±	62		175	±	51	
	CH	159	±	45	*	142	±	42	*	54	±	30		59	±	31		64	±	25		70	±	35		202	±	64	*	241	±	52	*
	M	172	±	56		168	±	48		168	±	48		173	±	48		179	±	48		183	±	53		177	±	61		175	±	66	
	MH	186	±	84	*	182	±	87	*	67	±	57		73	±	61		76	±	64		87	±	72		224	±	67	*	256	±	62	*
oral μvel [aU]	C	29	±	5		28	±	6		28	±	7		28	±	7		28	±	7		29	±	7		29	±	8		29	±	7	
	CH	26	±	5	*	25	±	5	*	17	±	5		17	±	5		18	±	4		18	±	5		31	±	7	*	35	±	6	*
	M	31	±	12		31	±	13		31	±	12		31	±	11		31	±	9		31	±	9		30	±	9		30	±	9	
	MH	29	±	12	*	30	±	11	*	19	±	9		20	±	9		20	±	9		21	±	10		34	±	10	*	38	±	10	*

Microcirculatory variables of the experimental groups—gastric and oral mucosal hemoglobin oxygenation (μHbO_2_); gastric and oral mucosal microcirculatory perfusion (μflow) and perfusion velocity (μvel). Data are presented as absolute values, mean ± SD, n = 6, *p < 0.05 vs. NV/HV30, 2-way ANOVA for repeated measurements followed by Dunnett's post hoc test; #p < 0.05 vs. respective control group (vs. C for M, vs. CH for MH). 2-way ANOVA for repeated measurements followed by Sidak post hoc test. BL, baseline; PS, preshock; NV/HV30/35/40/60 = 30/35/40/60 min after shock induction or the respective time control, RT30/60 = retransfusion.

**Table 2:** Systemic hemodynamic.

**Parameter**	**Group**	**BL**				**PS**				**NV/HV30**				**NV/HV35**				**NV/HV40**				**NV/HV60**				**RT30**				**RT60**			
MAP [mmHg]	C	60	±	4	*	63	±	5		64	±	5		63	±	5		63	±	4		63	±	4		64	±	6		64	±	7	
	CH	58	±	2	*	61	±	5	*	49	±	3		51	±	4		53	±	4		56	±	5	*	71	±	6	*	64	±	5	*
	M	61	±	4		62	±	3		62	±	3		62	±	3		62	±	3		62	±	3		63	±	4		62	±	5	
	MH	63	±	6	#*	63	±	3	*	48	±	2		50	±	6		51	±	5		56	±	3	*	71	±	5	*	67	±	5	*
HR [min^−1^]	C	116	±	9		117	±	11		115	±	11		114	±	11		114	±	11		114	±	11		112	±	11		109	±	12	
	CH	117	±	8		115	±	6		117	±	13		140	±	60		141	±	61		120	±	13		107	±	6		107	±	6	
	M	115	±	12		113	±	9		113	±	9		112	±	10		112	±	10		112	±	10		112	±	9		109	±	10	
	MH	116	±	7		114	±	5		115	±	12		137	±	60		138	±	60		119	±	11		105	±	5		107	±	5	
CO [ml·kg^−1^·min^−1^]	C	80	±	12		79	±	11		77	±	8										75	±	7		76	±	8		77	±	9	
	CH	82	±	10	*	85	±	17	*	46	±	2										53	±	3		81	±	10	*	84	±	10	*
	M	79	±	13		78	±	12		77	±	9										76	±	9		76	±	9		77	±	10	
	MH	78	±	9	*	77	±	8	*	45	±	7										52	±	5	*	79	±	9	*	82	±	10	*
DO_2_ [ml·kg^−1^·min^−1^]	C	12	±	3		12	±	3		11	±	2										11	±	2		11	±	2		11	±	2	
	CH	12	±	3	*	13	±	3	*	7	±	1										8	±	1		12	±	2	*	12	±	3	*
	M	12	±	3		11	±	3		11	±	2										11	±	2		11	±	2		11	±	2	
	MH	12	±	3	*	12	±	3	*	7	±	2										8	±	1		12	±	3	*	12	±	3	*
SVR [mmHg·l^−1^·min]	C	25	±	5		27	±	6		28	±	5										28	±	5		28	±	6		27	±	5	
	CH	24	±	4	*	24	±	6	*	35	±	5										35	±	5		29	±	4	*	25	±	4	*
	M	22	±	6		22	±	6		21	±	5										21	±	5		21	±	5		22	±	6	
	MH	26	±	5	*	27	±	5	*	35	±	6										35	±	5		30	±	5	*	27	±	4	*
SV [ml]	C	22	±	5		21	±	5		21	±	5										21	±	4		21	±	4		22	±	4	
	CH	22	±	4	*	23	±	6	*	12	±	2										14	±	3		23	±	3	*	24	±	4	*
	M	22	±	6		22	±	6		21	±	5										21	±	5		21	±	5		22	±	6	
	MH	21	±	4	*	21	±	4	*	12	±	2										14	±	2		24	±	4	*	24	±	5	*

Systemic hemodynamic variables of the experimental groups—mean arterial pressure (MAP), heart rate (HR), cardiac output (CO), systemic oxygen delivery (DO_2_), systemic vascular resistance (SVR) and stroke volume (SV). Data are presented as absolute values, mean ± SD, n = 6, *p < 0.05 vs. NV/HV30, 2-way ANOVA for repeated measurements followed by Dunnett's post hoc test; #p < 0.05 vs. respective control group (vs. C for M, vs. CH for MH). 2-way ANOVA for repeated measurements followed by Sidak post hoc test. Due to occasionally missing values of heart rate, no statistical analysis was performed. BL, baseline; PS, preshock; NV/HV30/35/40/60 = 30/35/40/60 min after shock induction or the respective time control, RT30/60 = retransfusion.

**Table 3:** Blood gas analysis and metabolic variables.

**Parameter**	**Group**	**BL**				**PS**				**NV/HV30**				**NV/HV60**				**RT30**				**RT60**			
SAT [%]	C	98.8	±	1.0		98.8	±	0.9		98.9	±	0.9		98.8	±	0.9		98.8	±	0.9		98.9	±	0.9	
	CH	98.3	±	1.1	*	98.5	±	0.9	*	97.6	±	1.4		98.0	±	1.2	*	98.6	±	0.8	*	98.6	±	0.8	*
	M	99.0	±	0.8	**#**	99.0	±	0.8	**#**	99.0	±	0.7	**#**	99.0	±	0.8	**#**	99.0	±	0.7	**#**	99.0	±	0.7	**#**
	MH	98.8	±	1.0	#*	98.8	±	1.0	*	98.2	±	1.6	**#**	98.4	±	1.4	**#**	98.9	±	0.9	*	98.8	±	0.9	*
pCO_2_ [mmHg]	C	37.4	±	2.5		37.1	±	2.7		37.4	±	2.3		37.9	±	2.2		37.9	±	1.8		38.8	±	3.4	*
	CH	36.7	±	2.6	*	36.6	±	2.1	*	41.5	±	3.1		40.2	±	2.5	*	36.8	±	1.9	*	36.8	±	1.9	*
	M	36.6	±	2.6		36.7	±	3.0		37.0	±	2.7		37.3	±	2.5		37.3	±	2.1		37.8	±	3.2	
	MH	36.6	±	2.4	*	36.3	±	2.4	*	41.1	±	3.9		40.0	±	4.1		36.7	±	2.4	*	37.3	±	2.2	*
pO_2_ [mmHg]	C	139.8	±	11.0	*	143.2	±	11.1		146.0	±	10.7		145.2	±	9.2		146.3	±	11.3		144.9	±	8.3	
	CH	139.0	±	14.2	*	142.5	±	10.6	*	128.0	±	10.5		136.5	±	9.9	*	148.8	±	10.9	*	149.2	±	10.5	*
	M	141.7	±	8.3		144.2	±	6.9		145.2	±	7.6		145.7	±	5.5		147.0	±	6.2		147.8	±	9.6	
	MH	143.2	±	11.2	*	144.0	±	9.0	*	131.5	±	10.3		137.5	±	10.4	*	152.5	±	8.9	*	148.2	±	7.8	*
pH	C	7.38	±	0.03		7.38	±	0.03		7.38	±	0.02		7.38	±	0.02		7.38	±	0.03		7.37	±	0.03	
	CH	7.38	±	0.03	*	7.38	±	0.03	*	7.31	±	0.03		7.32	±	0.03	*	7.37	±	0.02	*	7.37	±	0.02	*
	M	7.38	±	0.02		7.38	±	0.03		7.38	±	0.02		7.38	±	0.02		7.38	±	0.02		7.37	±	0.03	
	MH	7.38	±	0.03	*	7.38	±	0.02	*	7.32	±	0.04		7.33	±	0.04	*	7.37	±	0.03	*	7.37	±	0.03	*
HCO_3_ [mmol·l^−1^]	C	21.3	±	0.7		21.3	±	0.7		21.3	±	0.7		21.5	±	0.6		21.4	±	0.6		21.7	±	0.8	*
	CH	21.0	±	0.6	*	20.9	±	0.5	*	20.2	±	0.6		20.1	±	0.5		20.4	±	0.6		20.4	±	0.5	
	M	21.1	±	0.8		21.0	±	0.9		21.0	±	0.6		21.1	±	0.5		21.1	±	0.4		21.1	±	0.5	**#**
	MH	21.1	±	0.7	*	20.9	±	0.7	*	20.4	±	0.7		20.3	±	0.7		20.5	±	0.8		20.7	±	0.8	
Hb [g·100 ml^−1^]	C	11.1	±	1.1		11.0	±	1.1		10.9	±	1.0		10.8	±	1.1		10.9	±	1.2		10.8	±	1.3	
	CH	10.9	±	1.3		10.8	±	1.4		10.8	±	1.3		10.5	±	1.2	*	10.4	±	1.2	*	10.4	±	1.4	*
	M	10.7	±	1.2	**#**	10.5	±	1.3	**#**	10.5	±	1.3	**#**	10.4	±	1.3	**#**	10.5	±	1.3	**#**	10.4	±	1.3	**#**
	MH	10.9	±	1.4		10.8	±	1.5		11.0	±	1.4	**#**	10.8	±	1.2	**#**	10.6	±	1.3	#*	10.6	±	1.4	#*
lactate [mmol·l^−1^]	C	0.9	±	0.4		1.0	±	0.4		1.1	±	0.4		0.9	±	0.3		0.9	±	0.3		0.9	±	0.3	
	CH	0.9	±	0.3		1.1	±	0.4		1.4	±	0.3		1.2	±	0.3		1.0	±	0.3		1.0	±	0.3	
	M	1.0	±	0.4		1.1	±	0.4		1.2	±	0.4		1.1	±	0.3		1.1	±	0.3		1.1	±	0.3	
	MH	0.8	±	0.2		0.9	±	0.3		1.2	±	0.3		1.0	±	0.3		0.8	±	0.2		0.8	±	0.2	
BE [mmol·l^−1^]	C	−3.0	±	1.2		−2.9	±	0.8		−3.0	±	0.9		−2.8	±	0.9		−2.9	±	1.0		−2.7	±	0.7	
	CH	−3.0	±	0.8	*	−3.2	±	0.7	*	−4.9	±	0.7		−4.8	±	0.8		−3.8	±	0.8	*	−3.8	±	0.6	*
	M	−2.9	±	0.8		−3.1	±	0.8		−3.1	±	0.6		−3.1	±	0.5		−3.0	±	0.5		−3.1	±	0.5	
	MH	−2.9	±	0.9	*	−3.1	±	0.8	*	−4.6	±	0.8		−4.5	±	0.9		−3.7	±	1.0	*	−3.5	±	1.0	*

Metabolic variables of the experimental groups—arterial oxygen saturation (SAT), arterial carbon dioxide partial pressure (PaCO_2_), arterial oxygen partial pressure (P_a_O_2_), pH, bicarbonate (HCO_3_), hemoglobin concentration (Hb), lactate plasma concentration, and base excess (BE). Data are presented as absolute values, mean ± SD, n = 6, ^*^p < 0.05 vs. NV/HV30, 2-way ANOVA for repeated measurements followed by Dunnett's post hoc test; #p < 0.05 vs. respective control group (vs. C for M, vs. CH for MH). 2-way ANOVA for repeated measurements followed by Sidak post hoc test. Due to occasionally missing values of lactate plasma concentration, no statistical analysis was performed. BL, baseline; PS, preshock; NV/HV30/60 = 30/60 min after shock induction or the respective time control, RT30/60 = retransfusion.

### Effects of Melatonin During Hemorrhage and Retransfusion (Groups CH and MH)

#### Gastric Microcirculation

**Figure 2:**
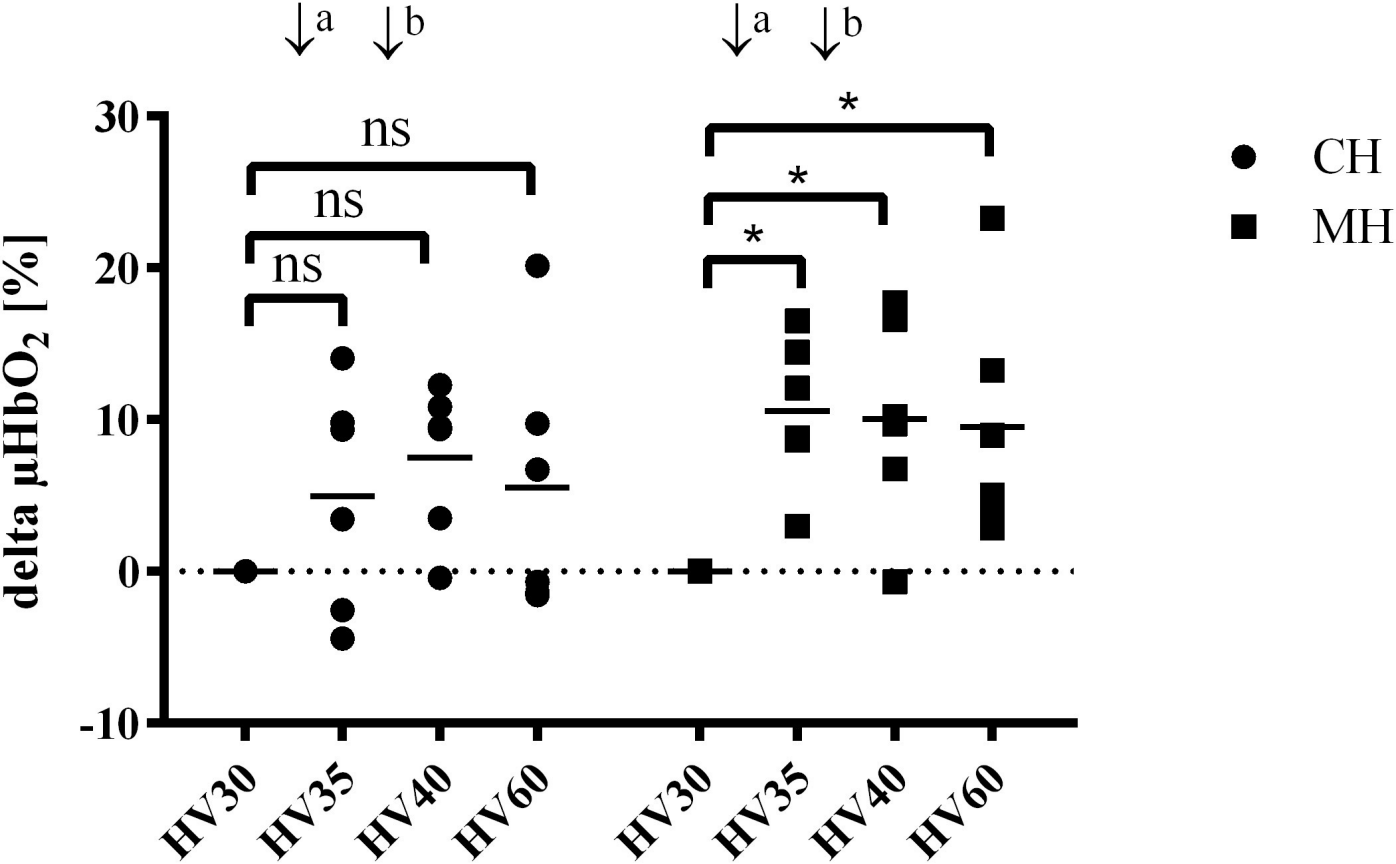
Δ gastric μHbO_2_ during hemorrhagic shock (hemorrhagic groups). Changes in gastric microcirculatory oxygenation (delta μHbO_2_) compared to the pretreament value (HV30) in anesthetized dogs during hemorrhagic shock with topical application (↓) of vehicle (CH, ↓^a^ NaCl 0.9%; ↓^b^ ethanol 5%) or melatonin (MH; ↓^a^ 0.3 mg; ↓^b^ 3 mg). Data are presented as individual values and mean for *n* = 6 dogs; *p < 0.05 vs. HV30, 2-way ANOVA for repeated measurements followed by Dunnett's *post hoc* test. HV30/35/40/60 = 30/35/40/60 min after shock induction.

Hemorrhagic shock reduced gastric μHbO_2_ from 80 ± 8 to 51 ± 14% (group CH) and from 81 ± 8 to 50 ± 15% (group MH) (Table 1). In the control group μHbO_2_ remained depressed after 60 min of shock. In contrast, melatonin application led to a significant increase in μHbO_2_ (HV35: 60 ± 13%; HV40: 60 ± 16%) compared to HV30 immediately before the local treatment. This increase sustained throughout the remaining state of shock (Figure 2). No significant differences to the control group were observed. After retransfusion of the shed blood μHbO_2_ was restored in both groups to 79 ± 9% (group CH) and 83 ± 6% (group MH) without differences between the groups. Gastric μflow was reduced during hemorrhage from 98 ± 21 to 69 ± 7 aU (group CH) and from 118 ± 33 to 83 ± 36 aU (group MH) (Table 1). The velocity of gastric microcirculatory perfusion (μvel) was higher in group MH compared to the control group during baseline conditions. After retransfusion μvel was higher in group MH compared to CH, similar to the significantly higher starting level (Table 1). Topical application of melatonin did not modulate local gastric perfusion.

#### Oral Microcirculation

During hemorrhagic shock, oral μHbO_2_ decreased from 85 ± 5 to 48 ± 2% (group CH) and from 84 ± 6 to 47 ± 3% (group MH) (Table 1). Oral μHbO_2_ showed an early recovery in the control group (HV35: 54 ± 5%) and melatonin group (HV40: 54 ± 10%). During the subsequent shock period oral μHbO_2_ recovered to 57 ± 7% in both groups (group CH and MH). In contrast to gastric microcirculation, extent of the recovery in microcirculatory oxygenation were not modulated by melatonin. During hemorrhage, oral μflow and μvelo were depressed in both groups. In parallel to the findings at the gastric mucosa, topical application of melatonin had no effect on oral microcirculatory blood flow and velocity (Table 1).

#### Systemic Hemodynamic Variables

**Figure 3:**
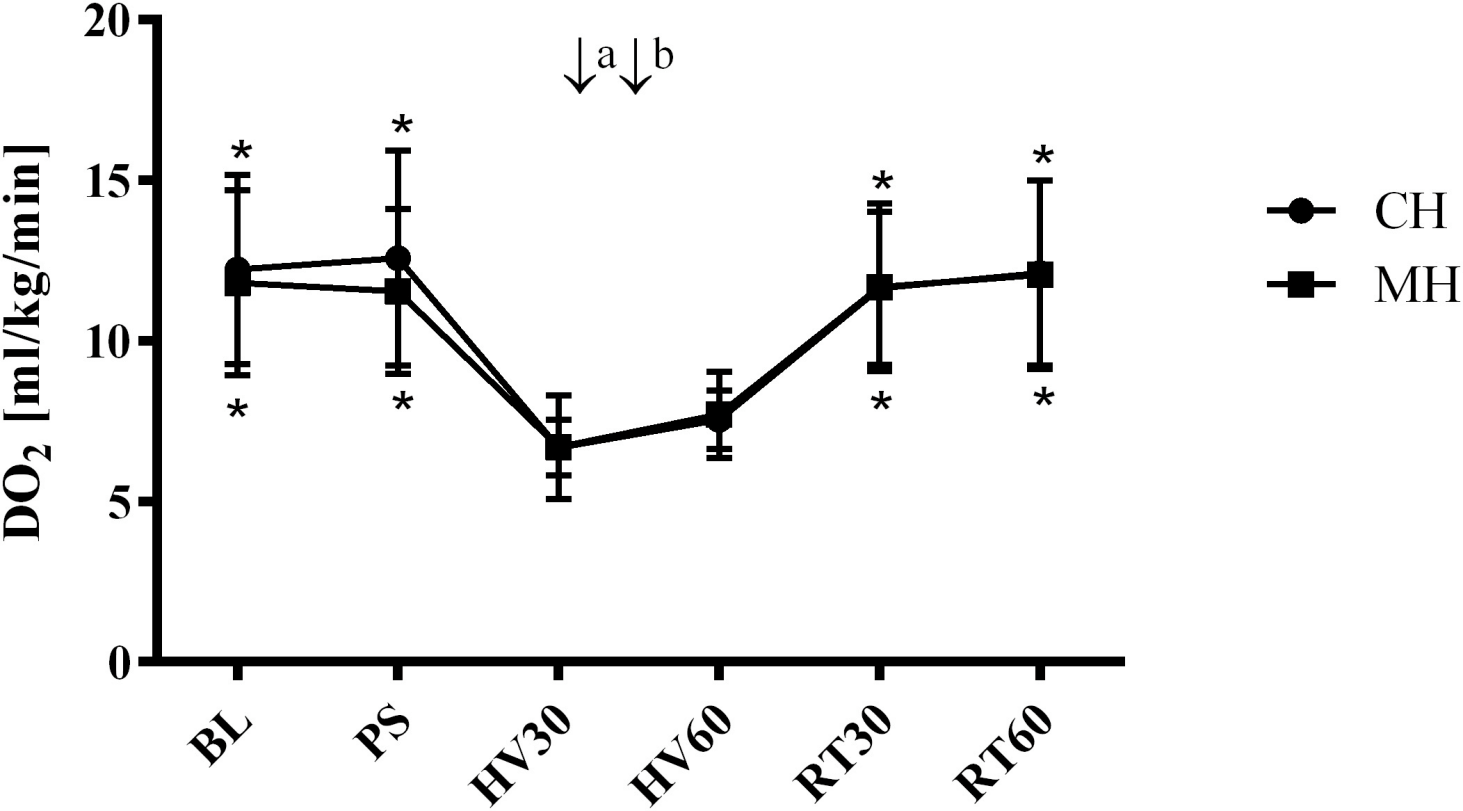
Systemic oxygen delivery (hemorrhagic groups). Systemic arterial oxygen delivery (DO_2_) in anesthetized dogs during hemorrhagic shock with topical application (↓) of vehicle (CH, ↓^a^ NaCl 0.9%; ↓^b^ ethanol 5%) or melatonin (MH; ↓^a^ 0.3 mg; ↓^b^ 3 mg). Data are presented as mean ± SD for *n* = 6 dogs; *p < 0.05 vs. HV30, 2-way ANOVA for repeated measurements followed by Dunnett's *post hoc* test. BL, baseline; PS, preshock; HV30/60 = 30/60 min after shock induction, RT30/60 = retransfusion.

DO_2_ decreased during hemorrhage equally in both groups from 12 ± 3 min^−1^ to 7 ± 1 ml kg^−1^ min^−1^ (group CH) and from 12 ± 3 to 7 ± 2 ml kg^−1^ min^−1^ and was restored after retransfusion to 12 ± 3 ml kg^−1^ min^−1^ in both groups (Figure 3). The decrease of DO_2_ is based on a similar decrease of cardiac output and an increase in SVR. Application of melatonin did not alter systemic hemodynamic variables during the subsequent 30 min of shock and following retransfusion period (Table 2). A metabolic acidosis evolved during hemorrhage, without differences between the melatonin treated group and the control group. Further metabolic variables are displayed in Table 3.

### Melatonin Plasma Levels

**Figure 4:**
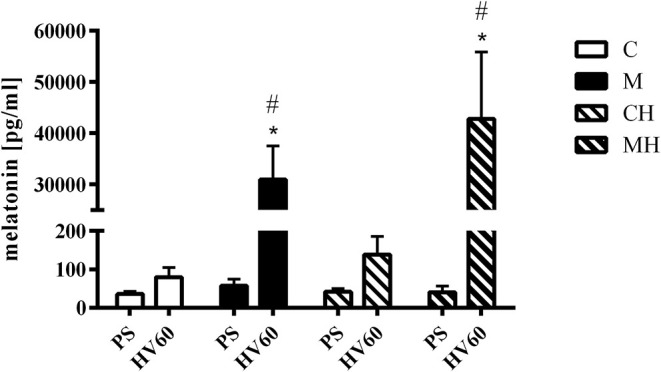
Melatonin plasma concentration. Melatonin plasma concentrations during baseline conditions and 30 min after topical application of the vehicle (C, CH) or melatonin (M, MH). Data are presented as mean ± SD for n = 6 dogs; *p < 0.05 vs. basic value (PS), #*p* < 0.05 vs. respective control group (vs. C for M, vs. CH for MH), 2-way ANOVA for repeated measurements followed by Sidak *post hoc* test. PS, preshock; HV60 = 60 min after shock induction.

Plasma melatonin levels remained within the physiological range during baseline conditions in all groups. During the course of the experiment no significant fluctuation in melatonin plasma concentration was seen in the control groups. Melatonin plasma concentration increased significantly to supraphysiological levels in melatonin treated animals (Figure 4).

### Effect of Melatonin on Caco-2 Cell Layer Integrity

**Figure 5:**
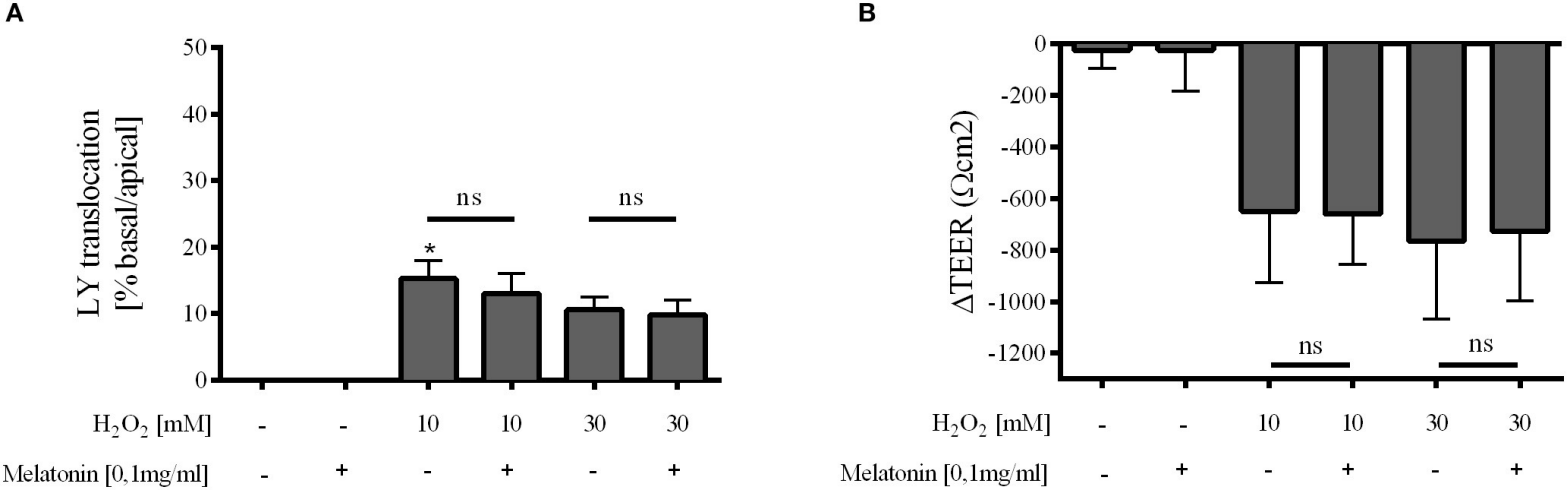
Caco-2 monolayer integrity. Relative translocation of LY **(A)** and transepithelial electrical resistance (TEER) **(B)** over the epithelial barrier from the apical to the basolateral compartment after co-incubation with melatonin 0.1 mg/ml and H_2_O_2_ 10 and 30 mM compared to the control group. Data shown as mean ± SD. *p < 0.05 vs. control, ns, not significant, *n* = 3, Kruskal-Wallis test with Dunn's multiple comparison test.

Melatonin showed no effect on Caco-2 cell layer integrity under physiologic conditions. Oxidative stress by incubation of the monolayer with 10 mM H_2_O_2_ let to a pronounced decline in cellular integrity as indicated by increased paracellular passage of LY and diminished TEER (Figure 5). The decrease in TEER was obvious but due to large standard deviation not significant. Melatonin had no direct effect on Caco-2 cell layer integrity, neither with pre-incubation prior to oxidative stress (Supplementary Figure 2) nor when co-incubated with H_2_O_2_ (Figure 5).

## Discussion

The main aim of the present study was to analyze the effects of topically applied melatonin on gastric and oral mucosal microcirculation during a mild hemorrhagic shock *in vivo* and the direct impact of melatonin on mucosal barrier integrity *in vitro*. Here, we could show that application of melatonin during a hemorrhagic shock may improve gastric microcirculatory oxygenation in dogs. Incubation of a CaCo-2 monolayer with melatonin did not directly influence mucosal barrier integrity.

In the *in vivo* part of this study we investigated the impact of melatonin on mucosal microcirculation using 0.3 mg melatonin solved NaCl 0.9% followed 5 min later by 3 mg melatonin solved in 5% ethanol. The chosen concentration is based on our previous *in vivo* study in dogs (7) and corresponds to ~100 μg kg^−1^ which has been shown to be protective in various settings in different species (20). The observed effects on gastric μHbO_2_ are independent of the solvent and 3 mg melatonin did not further increase gastric μHbO_2_. However, the study was not designed to compare different drug concentrations.

During physiological conditions in normovolemic anesthetized dogs, melatonin had no effect on micro- and macrohemodynamic variables. In contrast, melatonin was able to improve gastric microcirculatory oxygenation when applied topically to the gastric mucosa during hemorrhagic shock. The observed effect on gastric μHbO_2_ is rather small. Whereas, in the control group gastric μHbO_2_ remained depressed during the shock period, melatonin application enhanced gastric μHbO_2_ recovery during hemorrhage compared to the state of shock immediately before local treatment. These results are quite interesting and of potential clinical importance. Improving microcirculatory perfusion might fall short in improving local tissue function, as enhanced perfusion does not necessarily ensure sufficient oxygenation. Concerning gut integrity especially microcirculatory oxygenation is a major determinant for anastomotic healing (21). In a previous study we could demonstrate that an improved gastric μHbO_2_ by pretreatment with nitroglycerin prior to a hemorrhagic shock was associated with an ameliorated, shock induced damage to the gastric mucosal barrier (22). In this study, regional mucosal oxygenation was improved but regional perfusion remained unchanged. This observation contrasts with other reports on melatonin's effect on microcirculatory perfusion. Pretreatment or therapy with melatonin was able to improve hepatic and gastric perfusion in the context of a hemorrhagic shock in rats (23, 24) and dogs (7). However, in the present study we choose a regime with topical application of melatonin as a treatment during hemorrhagic shock. The regional gastrointestinal perfusion is mainly regulated at the level of the mesenteric arterioles (25). A pronounced vasoconstriction in these arterioles upstream the mucosal microcirculation, as part of the sympathetic mediated counter regulation to acute blood loss, may attenuate a potential melatonin mediated vasodilation in the capillary bed. As the systemic oxygen delivery and local gastric perfusion are not improved by melatonin, our results indicate, that the increased microcirculatory oxygenation did not result from improved local oxygen supply. Hence, μHbO_2_ increases due to a reduced oxygen demand or a cellular inability to extract oxygen. An oxygen extraction deficit seems to be unlikely, as melatonin is known to exert tissue protective effects even during conditions where oxygen delivery is impaired. More to the contrary, melatonin treatment has been shown to evoke a rightward shift of the oxygen dissociation curve of hemoglobin, thereby promoting tissue oxygenation. This was demonstrated in a rat model exposed to hypothermia (26). Whether similar effects may provide protective effects in the context of hemorrhage has to be studied in further detail. On the other hand, melatonin may improve mitochondrial respiration during pathologic conditions like hemorrhagic shock by inhibition of oxidative stress-induced mitochondrial damage (27). Furthermore, melatonin increased the activity of the mitochondrial complexes I and IV in brain and liver mitochondria, the only two tissues studied (28). Therefore, modulation of mitochondrial function may account for the increased postcapillary μHbO_2_ observed in the present study. While melatonin plasma concentrations during baseline and control conditions were similar to those described in different studies in various species (7, 29–31), local melatonin application led to a pronounced increase in plasma levels. We can only speculate, whether the observed effects on gastric μHbO_2_ are mediated via local mucosal or systemic effects of melatonin. The melatonin plasma levels measured 25 min after mucosal application corresponds to ~30–50% of the plasma concentrations after systemic melatonin application (7). However, as the topical melatonin application led to a modulation of gastric μHbO_2_ within minutes, a direct effect on local mucosal microcirculation is plausible.

Melatonin's microcirculatory protective effects are restricted to the gastric mucosa. A similar treatment of the oral mucosa showed inconclusive results, as oral μHbO_2_ improved after the second melatonin dose, but increased early in the control group, too. These observations are well in line with our previous results, indicating that the oral mucosa reacts differently compared to the gastric mucosa when treated with various vasoactive drugs (22, 32). At both locations, the verum was applied in direct proximity to the measuring site of the O2C device. During sevoflurane anesthesia the gastric motility is decreased, and gastric emptying is markedly prolonged (33). Therefore, dog's stomach is considered to be a relatively closed system with a minor clearance of gastric content toward the small intestine. Compared to this, after bolus application to the oral mucosa a relevant distribution of the active ingredient is likely, causing a consecutive reduction in effective concentration. Furthermore, the different histologic structure of the oral and gastric mucosa with diverse composition of the local microcirculation may account to the varying effects of mucosal application of vasoactive drugs. Once more our results underline, that a focus on the readily accessible oral microcirculation may not be suitable for the interpretation of further aboral parts of the gastrointestinal mucosal microcirculation by all means.

In the *in vivo* part of this study a cross-over design was chosen to reduce the number of animals needed to reach a sufficient power. However, the experimental group was not perfectly homogenous, indicated by occasional significant differences in micro-and macrocirculatory variables at baseline and pre-shock conditions. Although the exact reason is unclear, numerous factors, i.e., age of the animals or differences in stress hormone levels, could be causative. Carry-over effects of the preceding treatment are improbable, as melatonin has a half-life time of 20 min (34) and in humans elevated plasma levels after intake of 1–5 mg melatonin normalized within 8 h (35). After a single application of melatonin, a long-lasting (i.e., over 3 weeks) modulation of protein expression seems to be unlikely.

In the *in vitro* part of our experiments we analyzed the direct effects of melatonin on mucosal barrier integrity using the well-established Caco-2 cell culture model. To roughly simulate the conditions of oxidative stress induced by shock and reperfusion, the cells were exposed to exogenous administration of H_2_O_2_. Powers and colleagues demonstrated a similar reaction pattern of alveolar macrophages to oxidative stress generated by shock and reperfusion *in vivo* and by H_2_O_2_ *in vitro* (36). In the present study melatonin had no effect on mucosal barrier function. Neither LY translocation nor transepithelial electrical resistance was modulated after exogenous melatonin application. This is rather astonishing taken into account the known tissue protective properties of melatonin. In isolated fish hepatocytes exposed to 50 μM H_2_O_2_, addition of 0.1 mg/ml melatonin was sufficient to prevent the accumulation of free radicals and reactive oxygen species and to ameliorate the antioxidant status through modulation of transcription factors, e.g., NF-κB, ERK/Akt (37). Large datasets exist concerning melatonin's hepatoprotective properties [see (20) for further information], usually with a melatonin pretreatment regime for a couple of days or sometimes even weeks in advance to the harmful stimulus. Studies concerning the protective effect on the gut are sparse. Long-term administration of oral melatonin was demonstrated to maintain duodenal mucosal permeability during ethanol-induced stress (38) and to reduce colitis induced elevated gut permeability (39), thus improving intestinal barrier function in chronic disease. In contrast to these long-term pretreatment protocols, the present study focused on short term effects. While melatonin's antioxidative characteristics might be effective in the presence of H_2_O_2_, its anti-inflammatory properties will be secondary in the cell culture model. Furthermore, it remains unclear whether melatonin's tissue protective effects are mainly attributed to anti-oxidative radical scavenging properties or to systemic receptor-mediated effects, e.g., on regional microcirculation. In our previous study an increase in μflow after i.v. melatonin application was associated with a preserved intestinal barrier function during hemorrhagic shock (7). The missing protective effect of melatonin in the *in vitro* model indicates that melatonin's protective effect on intestinal barrier function is possibly not mediated via direct effects on the intestinal mucosa, but perhaps via an improvement of local microcirculation. However, the experimental setup does not mimic a therapeutically approach with initiation of the treatment after onset of oxidative stress. Therefore, the pre- and co-incubation regime limits the transferability of our results to the clinical setting. In the present model, application of H_2_O_2_ led to a pronounced increase in LY translocation and reduction in TEER. No dose dependence could be observed in cells treated with 10 mM and 30 mM H_2_O_2_. As even 10 mM H_2_O_2_ could result in an irreversible damage of the barrier function (40), an extensive cell breakdown could counteract any melatonin mediated potentially protective effects. These limitations could possibly explain the observed lack of influence of melatonin on Caco-2 monolayer integrity.

### Clinical Impact

Application of melatonin during resuscitation improved survival in a rat and porcine model of polytrauma and hemorrhagic shock (41, 42). No microcirculatory variables were assessed in these studies. However, a subsequent investigation revealed an increased urinary output in the first hours of resuscitation in melatonin treated pigs after trauma/hemorrhage (43). This could be related to an improved renal function. One might speculate, that an improved renal microcirculation during resuscitation enhanced renal recovery. Similar to our results, melatonin application had no effect on macrohemodynamic variables. However, melatonin application in these studies resulted in plasma concentrations of 8–10 mM, which are several orders of magnitude higher than the plasma levels observed in our present study (~17 nM). Therefore, application of higher melatonin concentrations may result in more pronounced microcirculatory effects. This has to be investigated in the future. Together with the results of our previous study (7) our present results confirm, that exogenous melatonin ameliorates gastric microcirculatory derangements during hemorrhagic shock. Beside the well-known anti-inflammatory and anti-oxidative properties, modulation of the microcirculation could be a new aspect of melatonin's known tissue protective effects. Several tissues including the intestine have been shown to express melatonin receptors (44) and the antioxidative effects of melatonin are potentially receptor mediated (20). Whether the melatonin-mediated attenuation of the microcirculation during hemorrhage and the modulation of mitochondrial function are receptor dependent has to be addressed in further studies.

## Data Availability Statement

All datasets presented in this study are included in the article/Supplementary Material.

## Ethics Statement

The animal study was reviewed and approved by North Rhine-Westphalia State Agency for Nature, Environment and Consumer Protection, Recklinghausen, Germany.

## Author Contributions

RT and IN: conception and design, acquisition of data, analysis and interpretation of data, and drafting the article. JS: analysis and interpretation of data and revising the article. AH: conception, analysis and interpretation of data, and revising the article. TH and LG: acquisition of data, analysis and interpretation of data, and revising the article. AR: conception and design, acquisition of data, analysis and interpretation of data, and revising the article. IB, OP, and CV: conception and design, analysis and interpretation of data, and revising the article. All authors read and approved the final manuscript.

## Conflict of Interest

The authors declare that the research was conducted in the absence of any commercial or financial relationships that could be construed as a potential conflict of interest.

## References

[1] Murray CJ Lopez AD. Alternative projections of mortality and disability by cause 1990-2020: Global Burden of Disease Study. Lancet Lond Engl. (1997) 349:1498–504. 10.1016/S0140-6736(96)07492-2 9167458

[2] Jakob SM Takala J. Gut perfusion in the critically ill. Intensive Care Med. (2000) 26:813–5. 10.1007/s001340051253 10945404

[3] Deitch EA Xu D Kaise VL. Role of the gut in the development of injury- and shock induced SIRS and MODS: the gut-lymph hypothesis, a review. Front Biosci J Virtual Libr. (2006) 11:520–8. 10.2741/1816 16146750

[4] Trzeciak S Dellinger RP Parrillo JE Guglielmi M Bajaj J Abate NL. Early microcirculatory perfusion derangements in patients with severe sepsis and septic shock: relationship to hemodynamics, oxygen transport, and survival. Ann Emerg Med. (2007) 49:88–98:98.e1–2. 10.1016/j.annemergmed.2006.08.021 17095120

[5] Chierego M Verdant C De Backer D. Microcirculatory alterations in critically ill patients. Minerva Anestesiol. (2006) 72:199–205. 16570031

[6] Brzozowski T Konturek PC Zwirska-Korczala K Konturek SJ Brzozowska I Drozdowicz D. Importance of the pineal gland, endogenous prostaglandins and sensory nerves in the gastroprotective actions of central and peripheral melatonin against stress-induced damage. J Pineal Res. (2005) 39:375–85. 10.1111/j.1600-079X.2005.00264.x 16207293

[7] Vollmer C Weber APM Wallenfang M Hoffmann T Mettler-Altmann T Truse R. Melatonin pretreatment improves gastric mucosal blood flow and maintains intestinal barrier function during hemorrhagic shock in dogs. Microcirc N Y N. (1994) 24:e12345. 10.1111/micc.12345 28316127

[8] Dyson DH. Positive pressure ventilation during anesthesia in dogs: assessment of surface area derived tidal volume. Can Vet J Rev Vét Can. (2012) 53:63–6. 22753965 PMC3239150

[9] Krug A CME: Mikrozirkulation und Sauerstoffversorgung des Gewebes - Methode des so genannten O2C (oxygen to see). Phlebologie. (2006) 35:300–12. 10.1055/s-0037-1622158

[10] Siegemund M van Bommel J Ince C. Assessment of regional tissue oxygenation. Intensive Care Med. (1999) 25:1044–60. 10.1007/s001340051011 10551958

[11] Temmesfeld-Wollbrück B Szalay A Mayer K Olschewski H Seeger W Grimminger F. Abnormalities of gastric mucosal oxygenation in septic shock: partial responsiveness to dopexamine. Am J Respir Crit Care Med. (1998) 157(5 Pt 1):1586–92. 10.1164/ajrccm.157.5.9710017 9603142

[12] Mythen MG Purdy G Mackie IJ McNally T Webb AR Machin SJ. Postoperative multiple organ dysfunction syndrome associated with gut mucosal hypoperfusion, increased neutrophil degranulation and C1-esterase inhibitor depletion. Br J Anaesth. (1993) 71:858–63. 10.1093/bja/71.6.858 8280554

[13] Fournell A Schwarte LA Kindgen-Milles D Müller E Scheeren TWL. Assessment of microvascular oxygen saturation in gastric mucosa in volunteers breathing continuous positive airway pressure. Crit Care Med. (2003) 31:1705–10. 10.1097/01.CCM.0000063281.47070.53 12794408

[14] Sato N Kawano S Kamada T Takeda M. Hemodynamics of the gastric mucosa and gastric ulceration in rats and in patients with gastric ulcer. Dig Dis Sci. (1986) 31 (2 Suppl.):35S−41S. 10.1007/BF01309321 3943456

[15] Kortbeek JB Al Turki SA Ali J Antoine JA Bouillon B Brasel K. Advanced trauma life support, 8th edition, the evidence for change. J Trauma. (2008) 64:1638–50. 10.1097/ta.0b013e3181744b03 18545134

[16] Herminghaus A Eberhardt R Truse R Schulz J Bauer I Picker O. Nitroglycerin and iloprost improve mitochondrial function in colon homogenate without altering the barrier integrity of caco-2 monolayers. Front Med. (2018) 5:291. 10.3389/fmed.2018.00291 30460235 PMC6232762

[17] Hidalgo IJ Raub TJ Borchardt RT. Characterization of the human colon carcinoma cell line (Caco-2) as a model system for intestinal epithelial permeability. Gastroenterology. (1989) 96:736–49. 10.1016/S0016-5085(89)80072-1 2914637

[18] Speckmann B Pinto A Winter M Förster I Sies H Steinbrenner H. Proinflammatory cytokines down-regulate intestinal selenoprotein P biosynthesis via NOS2 induction. Free Radic Biol Med. (2010) 49:777–85. 10.1016/j.freeradbiomed.2010.05.035 20542496

[19] Feighery LM Cochrane SW Quinn T Baird AW O'Toole D Owens S-E. Myosin light chain kinase inhibition: correction of increased intestinal epithelial permeability in vitro. Pharm Res. (2008) 25:1377–86. 10.1007/s11095-007-9527-6 18163202

[20] Mathes AM. Hepatoprotective actions of melatonin: possible mediation by melatonin receptors. World J Gastroenterol. (2010) 16:6087–97. 10.3748/wjg.v16.i48.6087 21182223 PMC3012585

[21] Sheridan WG Lowndes RH Young HL. Tissue oxygen tension as a predictor of colonic anastomotic healing. Dis Colon Rectum. (1987) 30:867–71. 10.1007/BF02555426 3677962

[22] Truse R Hinterberg J Schulz J Herminghaus A Weber A Mettler-Altmann T. Effect of topical iloprost and nitroglycerin on gastric microcirculation and barrier function during hemorrhagic shock in dogs. J Vasc Res. (2017) 54:109–21. 10.1159/000464262 28441653

[23] Mathes AM Kubulus D Pradarutti S Bentley A Weiler J Wolf B. Melatonin pretreatment improves liver function and hepatic perfusion after hemorrhagic shock. Shock Augusta Ga. (2008) 29:112–8. 10.1097/shk.0b013e3180644ca3 17666950

[24] Mathes AM Kubulus D Weiler J Bentley A Waibel L Wolf B. Melatonin receptors mediate improvements of liver function but not of hepatic perfusion and integrity after hemorrhagic shock in rats. Crit Care Med. (2008) 36:24–9. 10.1097/01.CCM.0000292088.33318.F0 18090374

[25] Shah V Lyford G Gores G Farrugia G. Nitric oxide in gastrointestinal health and disease. Gastroenterology. (2004) 126:903–13. 10.1053/j.gastro.2003.11.046 14988844

[26] Hlutkin S Zinchuk V. Effect of melatonin on the blood oxygen transport during hypothermia and rewarming in rats. Adv Med Sci. (2008) 53:234–9. 10.2478/v10039-008-0035-7 18930873

[27] Reiter RJ Tan DX Manchester LC El-Sawi MR. Melatonin reduces oxidant damage and promotes mitochondrial respiration: implications for aging. Ann N Y Acad Sci. (2002) 959:238–50. 10.1111/j.1749-6632.2002.tb02096.x 11976199

[28] Martín M Macías M Escames G León J Acuña-Castroviejo D Melatonin but not vitamins C and E maintains glutathione homeostasis in t-butyl hydroperoxide-induced mitochondrial oxidative stress. FASEB J Off Publ Fed Am Soc Exp Biol. (2000) 14:1677–9. 10.1096/fj.99-0865fje 10973915

[29] Wehr TA Aeschbach D Duncan WC. Evidence for a biological dawn and dusk in the human circadian timing system. J Physiol. (2001) 535 (Pt 3):937–51. 10.1111/j.1469-7793.2001.t01-1-00937.x 11559786 PMC2278827

[30] Chuffa LGA Seiva FRF Fávaro WJ Teixeira GR Amorim JPA Mendes LO. Melatonin reduces LH, 17 beta-estradiol and induces differential regulation of sex steroid receptors in reproductive tissues during rat ovulation. Reprod Biol Endocrinol RBE. (2011) 9:108. 10.1186/1477-7827-9-108 21810236 PMC3161940

[31] Lazado CC Kumaratunga HPS Nagasawa K Babiak I Giannetto A Fernandes JMO. Daily rhythmicity of clock gene transcripts in atlantic cod fast skeletal muscle. PLoS ONE. (2014) 9:e99172. 10.1371/journal.pone.0099172 24921252 PMC4062345

[32] Truse R Voß F Herminghaus A Schulz J Weber A Mettler-Altmann T. Local gastric RAAS-inhibition improves gastric microvascular perfusion in dogs. J Endocrinol. (2019) 241:235–47. 10.1530/JOE-19-0030 30978701

[33] Boscan P Cochran S Monnet E Webb C Twedt D. Effect of prolonged general anesthesia with sevoflurane and laparoscopic surgery on gastric and small bowel propulsive motility and pH in dogs. Vet Anaesth Analg. (2014) 41:73–81. 10.1111/vaa.12093 24127667

[34] Claustrat B Brun J Chazot G. The basic physiology and pathophysiology of melatonin. Sleep Med Rev. (2005) 9:11–24. 10.1016/j.smrv.2004.08.001 15649735

[35] Tordjman S Chokron S Delorme R Charrier A Bellissant E Jaafari N. Melatonin: pharmacology, functions and therapeutic benefits. Curr Neuropharmacol. (2017) 15:434–43. 10.2174/1570159X14666161228122115 28503116 PMC5405617

[36] Powers KA Szászi K Khadaroo RG Tawadros PS Marshall JC Kapus A. Oxidative stress generated by hemorrhagic shock recruits Toll-like receptor 4 to the plasma membrane in macrophages. J Exp Med. (2006) 203:1951–61. 10.1084/jem.20060943 16847070 PMC2118368

[37] Moniruzzaman M Ghosal I Das D Chakraborty SB. Melatonin ameliorates H2O2-induced oxidative stress through modulation of Erk/Akt/NFkB pathway. Biol Res. (2018) 51:17. 10.1186/s40659-018-0168-5 29891016 PMC5996524

[38] Sommansson A Yamskova O Schiöth HB Nylander O Sjöblom M. Long-term oral melatonin administration reduces ethanol-induced increases in duodenal mucosal permeability and motility in rats. Acta Physiol Oxf Engl. (2014) 212:152–65. 10.1111/apha.12339 24995603

[39] Trivedi PP Jena GB. Melatonin reduces ulcerative colitis-associated local and systemic damage in mice: investigation on possible mechanisms. Dig Dis Sci. (2013) 58:3460–74. 10.1007/s10620-013-2831-6 23975342

[40] Catalioto R-M Festa C Triolo A Altamura M Maggi CA Giuliani S. Differential effect of ethanol and hydrogen peroxide on barrier function and prostaglandin E2 release in differentiated Caco-2 cells: selective prevention by growth factors. J Pharm Sci. (2009) 98:713–27. 10.1002/jps.21439 18481313

[41] Klein AH Wendroth SM Drewes LR Andrews MT. Small-volume d-β-hydroxybutyrate solution infusion increases survivability of lethal hemorrhagic shock in rats. Shock Augusta Ga. (2010) 34:565–72. 10.1097/SHK.0b013e3181e15063 20386494

[42] Mulier KE Lexcen DR Luzcek E Greenberg JJ Beilman GJ. Treatment with beta-hydroxybutyrate and melatonin is associated with improved survival in a porcine model of hemorrhagic shock. Resuscitation. (2012) 83:253–8. 10.1016/j.resuscitation.2011.08.003 21864484

[43] Wolf A Mulier KE Iyegha UP Asghar JI Beilman GJ. Safety of D-ß-hydroxybutyrate and melatonin for the treatment of hemorrhagic shock with polytrauma. Shock Augusta Ga. (2015) 44 (Suppl. 1):79–89. 10.1097/SHK.0000000000000315 25692249

[44] Sallinen P Saarela S Ilves M Vakkuri O Leppäluoto J. The expression of MT1 and MT2 melatonin receptor mRNA in several rat tissues. Life Sci. (2005) 76:1123–34. 10.1016/j.lfs.2004.08.016 15620576

