# Supplementary material for: Topical Melatonin Improves Gastric Microcirculatory Oxygenation During Hemorrhagic Shock in Dogs but Does Not Alter Barrier Integrity of Caco-2 Monolayers
Source: Front Med (Lausanne). 2020 Aug 28;7:510. doi: 10.3389/fmed.2020.00510 (PMC7484810; doi:10.3389/fmed.2020.00510)
Supplement: Supplementary file 1 [file Table_1.docx]

**Suppl. Data Tab. 1: dog’s biological data and sequence of experiments**

Age, body weight, shed blood volume and the chronological order of the four experimental groups (application of the vehicle (C) or melatonin (M) during physiological conditions or 30 min after induction of hemorrhagic shock (CH, MH)) for the six dogs (A – F).

| dog | age (years) | body weight (kg) | shed blood volume (ml)  (= 16 ml/kg) | order of experiments |
| --- | --- | --- | --- | --- |
| A | 6 | 36 | 576 | MH-CH-M-C |
| B | 9 | 28 | 448 | M-C-MH-CH |
| C | 9 | 29 | 464 | C-M-CH-MH |
| D | 3 | 32 | 512 | CH-C-M-MH |
| E | 9 | 32 | 512 | MH-CH-C-M |
| F | 7 | 28 | 448 | C-MH-CH-M |
